# Non-Invasive Micro-Test Technology in Plant Physiology Under Abiotic Stress: From Mechanism to Application

**DOI:** 10.3390/plants14131932

**Published:** 2025-06-23

**Authors:** Tianpeng Zhang, Peipei Yin, Xinghong Yang, Yunqi Liu, Ruirui Xu

**Affiliations:** 1 College of Biology and Oceanography, Weifang University, Weifang 261061, China; zhangtianpeng0718@163.com (T.Z.); yinpp1524@163.com (P.Y.); 2 State Key Laboratory of Wheat Improvement, College of Life Sciences, Shandong Agricultural University, Tai’an 271000, China; xhyang@sdau.edu.cn; 3 Zhongguancun Xuyue Non-Invasive Micro-Test Technology Industrial Alliance, Beijing 100080, China; yunqi@nmtia.org.cn

**Keywords:** Non-invasive Micro-test Technology, plant physiology, abiotic stress, ion transport, real-time assessment

## Abstract

Non-invasive Micro-test Technology (NMT) represents a pioneering approach in the study of physiological functions within living organisms. This technology possesses the remarkable capability to monitor the flow rates and three-dimensional movement directions of ions or molecules as they traverse the boundaries of living organisms without sample destruction. The advantages of NMT are multifaceted, encompassing real-time, non-invasive assessment, a wide array of detection indicators, and compatibility with diverse sample types. Consequently, it stands as one of the foremost tools in contemporary plant physiological research. This comprehensive review delves into the applications and research advancements of NMT within the field of plant abiotic stress physiology, including drought, salinity, extreme temperature, nutrient deficiency, ammonium toxicity, acid stress, and heavy metal toxicity. Furthermore, it offers a forward-looking perspective on the potential applications of NMT in plant physiology research, underscoring its unique capacity to monitor the flux dynamics of ions/molecules (e.g., Ca^2+^, H^+^, K^+^, and IAA) in real time, reveal early stress response signatures through micrometer-scale spatial resolution measurements, and elucidate stress adaptation mechanisms by quantifying bidirectional nutrient transport across root–soil interfaces. NMT enhances our understanding of the spatiotemporal patterns governing plant–environment interactions, providing deeper insights into the molecular mechanism of abiotic stress resilience.

## 1. Introduction

Plant adversity encompasses a broad array of environmental factors that are detrimental to plant growth and survival. Through extensive adaptation and evolution in nature, plants have acquired specific resilience and adaptability to harsh conditions, including extreme temperatures, drought, salinity, and heavy metal contamination [1,2,3]. Upon exposure to these environmental stressors, plants perceive and transmit environmental signals through multiple core physiological processes. These include regulating nutrient absorption via root membrane transporters, transducing stress signals through calcium and ROS waves, modulating stomatal aperture via guard cell ion dynamics, and coordinating developmental and stress adaptations through the auxin transport and signaling pathway [4,5,6]. These processes, operating at both organismal and cellular levels, elicit the coordinated transmission and regulation of endogenous and exogenous signals to maintain growth and metabolic homeostasis.

Non-invasive Micro-test Technology (NMT) is a key technology that uses selective microelectrodes to detect the ion and molecular transmembrane transport of living samples without touching or damaging them. NMT can monitor a wide range of samples in vivo, including bacteria, single cells, vacuoles, tissues, and organs. NMT has emerged as a pivotal tool in plant physiology research, particularly in elucidating ionic flux dynamics under abiotic stress conditions. Its adoption has generated paradigm-shifting discoveries in ionic dynamic analysis [7], stress signaling transduction [8], and root growth and development [9], as evidenced by publications in top-tier journals including *Science*, *Cell*, and *Nature*. NMT’s unique capability for non-invasive, real-time monitoring of micron-level plant materials can accurately capture ions’ spatial–temporal movement patterns in plant tissues [10]. This temporal resolution advantage, achieving detection in seconds, proves critical for capturing transient stress responses like rapid calcium signaling response and auxin transport during stress perception [11,12,13]. This technological advancement fosters a better understanding of nature through elucidating fundamental mechanisms underlying plant–environment interactions.

## 2. An Overview of NMT

NMT represents a diverse array of microelectrode technologies and methodologies. These methodologies include, but are not limited to, Scanning Ion-selective Electrode Technology (SIET), Scanning Vibrating Electrode Technique (SVET), Scanning Polarographic Electrode Technique (SPET), Self-Referencing Ion-Selective Electrode Technique (SERIS), Self-Referencing Polarographic Electrode Technique (SERP), Self-Referencing Enzyme-Assisted Electrode Technique (SERE), Scanning Reference Electrode Technique (SRET), and Microelectrode Ion Flux Estimation Technique (MIFE), alongside other pioneering technologies, as documented by Kunkel and Shabala [14,15].

The development of NMT can be traced back to the pioneering work of Lionel F. Jaffe from the Marine Biological Laboratory (MBL), who first introduced the initial concept in the 1970s. Ian A. Newman conducted the first experiment of H^+^ and K^+^ flux measurements in corn roots by using MIFE methodology in 1987 [16]. Subsequently, NMT was successfully employed to measure the Ca^2+^ flux rate of cells in 1990, addressing numerous scientific challenges [17,18]. In 1995, Smith, also from MBL, further elaborated on the physical principles, mathematical foundations, and application modes of NMT, significantly enhancing the theoretical framework of this technology [19]. By 2001, NMT achieved the capabilities of programmable sensor motion-tracking and three-bit automatic measurement, marking the emergence of modern NMT. The non-invasive micro-measurement system developed by Younger USA LLC (Amherst, MA, USA) and Xuyue (Beijing) Science and Technology Co., Ltd., (Beijing, China) currently represents the ninth generation of automated NMT products. This system received the “internationally leading” achievement evaluation from the Ministry of Science and Technology of China and has obtained certifications from the NMT Industry Alliance in Zhongguancun and ISO9001 quality assurance [20].

### 2.1. Technical Principles

**Figure 1:**
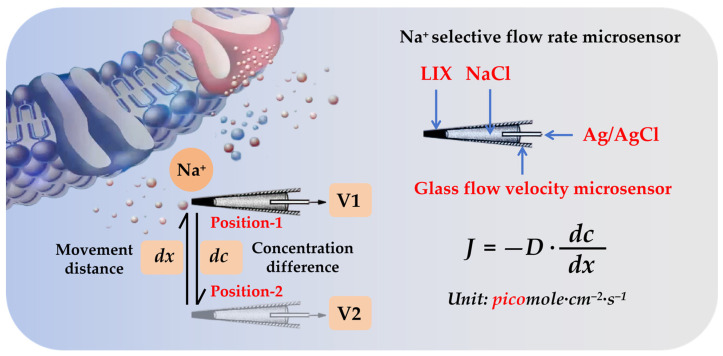
An illustration of the working principle of ion-selective flux rate microsensors in NMT, showing a case using Na^+^ concentration gradient and a Na^+^-selective flux rate microsensor. The Na^+^-selective flow rate microsensor achieves ion selectivity for Na^+^ by filling a Liquid Ion Exchanger (LIX) into its front-end tip. This flow rate microsensor measures the voltage (V_1_ and V_2_) at two points separated by a known distance (dx) within the concentration gradient of the target ion. The concentration difference (dc) between these two points can then be calculated from V_1_, V_2_, and the known voltage/concentration calibration curve of the microsensor (based on the Nernst Equation). D represents the ion’s diffusion constant (unit: cm^−2^·s^−1^). Substituting these values into Fick’s First Law of Diffusion (J = −D (dc/dx)) yields the flow rate of the ion (unit: pico mol·cm^−2^·s^−1^). This unit represents the number of moles of the ion/molecule passing through per square centimeter per second.

NMT utilizes flow-rate microsensors to capture the signals of ions and molecules. Based on the Nernst Equation and Fick’s First Law of Diffusion, it calculates the concentrations and flow rates of ions and molecules, enabling the detection of extremely subtle signals with flow rates reaching magnitudes as low as 10^−12^ mol·cm^−2^·s^−1^. The ion-selective microsensor of the NMT system comprises a glass microsensor, a Ag/AgCl wire, an electrolyte, and a liquid ion exchanger. It selectively recognizes specific ions via a class of organic compounds, specifically large neutral molecular carriers, within the liquid ion exchanger. Taking the measurement of the flux rate of Na^+^ as an illustrative example, the Na^+^-selective flux rate microsensor achieves selectivity for Na^+^ through the liquid ion exchanger (LIX) filled at its tip (Figure 1) [21]. The flux rate microsensor measures two points at a predefined distance, dx, within the ion concentration gradient to be assessed, yielding voltages V_1_ and V_2_, respectively. The concentration difference, dc, between these two points can be calculated using V_1_, V_2_, and the known voltage/concentration correction curve of the microsensor, which is based on the Nernst Equation. D denotes the diffusion constant of the ion (units: cm^−2^·s^−1^). Substituting these parameters into Fick’s First Law of Diffusion and the Nernst Equation (J = −D (dc/dx)) yields the flux rate of the ion (units: pico mol·cm^−2^·s^−1^), representing the number of moles passing through the ion/molecule per square centimeter per second [22,23]. It is worth noting that NMT calculates ion/molecule flux rates with a sensitivity of 10^−13^ mol·cm^−2^·s^−1^ for ions and 10^−15^ mol·cm^−2^·s^−1^ for molecules. The measurement distance between two sensor points is in the range of 5–50 μm, and NMT offers a time resolution of 4–6 s. Calibration can be achieved by solution preparation via dilution, and fluorescent dyes/optical fibers, carbon nanowires, enzyme electrodes, metals/alloys, and other materials can be utilized to carry out the selective measurement of certain ions/molecules.

### 2.2. Features, Advantages, and Application Fields

NMT is characterized by in vivo real-time measurements with high sensitivity (up to femtomoles cm^−2^ s^−1^) and spatial resolution (0.5–10 μm for ions, 2–25 μm for molecules). Its non-invasive nature allows for the simultaneous quantification of over 50 parameters, including Ca^2+^, H^+^, K^+^, Na^+^, NH_4_^+^, heavy metals (e.g., Cd^2+^), reactive oxygen species (e.g., H_2_O_2_), and phytohormones (e.g., IAA), without sample extraction [10,22,24]. One technical advantage is its unique three-dimensional gradient measurement capability based on Fick’s diffusion laws, enabling the dynamic tracking of ion/molecule fluxes across plant membranes under stress conditions [22,25].

While NMT has broad applications spanning biomedical and pharmacological research (e.g., neural activity detection and drug efficacy evaluation), its significant contributions to plant stress physiology merit particular emphasis. In plant physiology research, NMT has revolutionized our understanding of stress adaptation mechanisms through some key applications, such as ion homeostasis regulation, heavy metal detoxification, oxidative stress response, and auxin transport and signal transduction [10]. The real-time quantification of K^+^/Na^+^ flux dynamics in root cells under salinity stress revealed compartmentalization strategies in halophytes [26,27]. NMT’s high resolution enables the detection of H^+^-ATPase-driven proton gradients crucial for maintaining membrane potential under abiotic stress [28,29]. Furthermore, NMT’s multi-parameter capability (measuring two ions simultaneously) contributes to detecting the transmembrane ion flow rate and external ion concentration, and its high resolution can capture rapid signaling response events in the early stages of visible stress symptoms [10,22]. These developments facilitate the whole-plant analysis of ion redistribution patterns under abiotic stress treatments, significantly advancing our ability to cultivate stress-tolerant genotypes in crops.

## 3. NMT in Plant Physiology Under Abiotic Stress

The transport of mineral ions into and out of tissues and cells is central to the lives of plants [30]. Auxin plays a critical role in regulating plant adaptation and resilience to abiotic stresses and has agricultural potential for improving crop productivity [31]. NMT revolutionizes our understanding of plant stress response mechanisms by quantifying key ion and molecule fluxes. This includes quantifying ion homeostasis in real time by analyzing K^+^/Na^+^ transport kinetics under salinity stress [32], revealing stress signaling pathways by tracking the spatiotemporal patterns of Ca^2+^ waves under cold stress [13], and elucidating stomatal regulation by dynamically analyzing K^+^/NO_3_^−^ redistribution in guard cells and altering polar auxin transport in the root system under drought stress [12,33]. Thus, NMT offers a novel perspective by harnessing dynamic ion/molecular flux information in and out of plants, thereby unveiling more nuanced alterations in the physiological functions of diverse living plant samples. This technique provides a technological pathway for exploring plant functionalities and physiological mechanisms across multiple scales.

### 3.1. Salt Stress

Soil salinization poses an increasingly serious threat to agricultural and forestry productivity as well as environmental sustainability. It exerts diverse detrimental impacts on plants, encompassing ion toxicity, osmotic stress, and nutrient imbalances, ultimately hampering plant growth [34,35]. In response to a salt stress environment, plants leverage the transmembrane flux of ions across their cellular membranes. Sustaining an optimal K^+^/Na^+^ balance ratio is indispensable for plants to acclimate to high-salinity environments [36]. Tissue-specific K^+^ retention and ROS-specific regulation confer enhanced salinity tolerance in the halophyte quinoa. Tolerant accession specifically exhibited enhanced root plasma membrane integrity and improved K^+^ retention capacity in the mature root zone under saline stress [37]. NMT and molecular experiment results demonstrate that the superior leaf mesophyll K^+^ retention capacity in quinoa is conferred by intrinsically lower H^+^-ATPase activity, the reduced sensitivity of K^+^ transporters to ROS, and the enhanced sensitivity of ROS-activated Ca^2+^-permeable channels [38]. Similarly, NMT was employed to conduct a comparative analysis of the salt stress adaptation strategies between barley and triticale, two salt-tolerant cereal species. The results reveal that the desensitization of K^+^ and Ca^2+^-ROS-induced cation channels emerged as a key characteristic conferring salt tolerance. Compared with barley genotypes, triticale genotypes demonstrated more effective cytoplasmic K^+^ retention in the root elongation zone under salt stress conditions [39]. Calmodulin (CaM), a highly conserved calcium-binding protein, plays a crucial role in plants’ responses to salt stress. MIFE measurements revealed that, compared with WT plants, *OsCaM1-1* knockout mutants exhibited significantly higher Na^+^ concentrations and Na^+^/K^+^ ratios in both shoots and roots under salt stress, along with reduced transient K^+^ and Ca^2+^ fluxes in roots. These findings indicate that OsCaM1-1 positively regulates salt tolerance in rice by mediating Ca^2+^ signaling to maintain Na^+^ and K^+^ homeostasis [40].

The Salt Overly Sensitive (SOS) signaling module, consisting of the sodium transporter SOS1 and regulatory proteins SOS2 and SOS3, represents a well-established central mechanism for salt excretion, aiding plants in combating salt accumulation [5,41]. The findings demonstrate that the salt-induced calcium signal is decoded by 14-3-3 and SOS3/SCaBP8 proteins, which selectively activate/inactivate downstream protein kinases SOS2 and PKS5. These proteins regulate Na^+^ homeostasis by co-mediating the activity of the Na^+^/H^+^ antiporter and H^+^-ATPase in the plasma membrane. NMT was utilized to ascertain how PKS5 influences plant salt tolerance by monitoring the root Na^+^ flux in *Arabidopsis* [42]. Analogously, VPS23A constitutes an essential component of the internal separation complex required for transport, playing a crucial role in the functionality of the SOS module to confer salt tolerance to plants. The determination of the Na^+^ efflux rate at the *Arabidopsis* root tip via NMT unveiled that VPS23A positively impacts the secretion of Na^+^ in plants under high-salinity conditions and underscores the significance of SOS2 sorting in functioning at the cell membrane [41]. In the salt-tolerant woody species *Populus euphratica*, high salinity (200 mM NaCl) increases the transcription of phospholipase Dδ (PLDδ) in roots and stems. Using NMT and biochemical experiments it was confirmed that PeGLABRA3, a basic helix–loop–helix (bHLH) transcription factor, activates the transcription of *AtPLDδ* by binding to its promoter region under salt stress. This activation confers Na^+^ and ROS homeostasis through the PLDδ- and phosphatidic acid-mediated SOS1 signaling pathway, thereby enhancing salt adaptation in *Populus euphratica* [43].

In plants, glycine-rich RNA-binding proteins (GRPs) have been found to be expressed under various environmental stresses. Following long-term NaCl stress, PeGRP2 negatively affects mRNA stability. Using NMT to measure the Na^+^ efflux rate in the root meristem zone of *Populus euphratica* under salt stress, it was observed that NaCl significantly increased Na^+^ efflux in WT poplar. In transgenic lines, reduced Na^+^ efflux capacity led to the greater accumulation of salt in the roots [44]. The HKT-type proteins play vital roles in long-distance Na^+^ transport, maintenance of ion homeostasis, and improvement of salt tolerance in plants. In *Limonium bicolor*, by integrating NMT results, HKT-type proteins have been demonstrated to be involved in the salt secretion of salt glands for the first time, which provides a new perspective on the functions of HKT-type proteins under salt-stress conditions [45]. Regarding stress mitigation analysis, NMT revealed that exogenous methyl jasmonate (MeJA) promotes Na^+^ efflux in *Nitraria tangutorum* roots, reduces the Na^+^/K^+^ ratio, and mitigates salt stress [46]. Similarly, boron (B) is an essential micronutrient for plants and plays a significant role in alleviating the inhibitory effects of soil salt stress on plant growth. NMT revealed that exogenous B induces Cl^-^ efflux in roots under NaCl stress, providing critical evidence for exploring the mechanism of boron-mediated alleviation of salt toxicity in sugar beet [47]. Using MIFE, researchers investigated the real-time transport rates of K^+^ and H^+^ in barley roots under 200 mM of NaCl stress with boron supplementation. The results reveal that under B-sufficient conditions, both root and shoot Na^+^ contents were significantly reduced, while the K^+^ content increased, leading to an elevated K^+^/Na^+^ ratio. This physiological adjustment consequently enhanced barley’s tolerance to saline stress [48].

Autofluorescent inclusion (AFI), specifically accumulated in the mesophyll cells of non-secretory mangroves, is associated with salt adaptation. As a representative non-secretory mangrove species, *Kandelia obovata* exhibits exceptional salt tolerance. NMT and spatial metabolomics confirmed that AFI is structurally identified as condensed tannin (CT). CT biosynthesis showed a positive correlation with Na^+^ accumulation in leaves, where chloroplast-synthesized CT is cytoplasmically transported to vacuoles, acting as molecular carriers to facilitate the compartmentalized storage of excess Na^+^, thereby enhancing salt tolerance in *Kandelia obovata* [49]. Similarly, in studies on *Kandelia obovata* using NMT, it was confirmed that salicylic acid (SA)-induced salt tolerance relies on H_2_O_2_ generated by NADPH oxidases, which modulates Na^+^/K^+^ and redox homeostasis under high-salinity conditions [50]. In contrast, brassinosteroid (BR) enhances salt tolerance by reducing oxidative damage and regulating Na^+^/K^+^ homeostasis through the S-nitrosoglutathione reductase (GSNOR) and NO signaling pathways, thereby mediating the adaptation of *Kandelia obovata* to salt stress [51]. Integrated with NMT, elucidating the mechanisms of ion homeostasis regulation and osmoregulation in plants under salt stress will contribute to addressing the critical issues of soil salinity excess and Na^+^ toxicity in agricultural production and ecosystems (Table 1).

### 3.2. Alkali Stress

Excessive salt is detrimental to plant growth and development because high soil salinity levels lead to severe degradation of plant ecosystems and negatively impact agricultural productivity. Notably, soil salinity often co-occurs with alkalinity [52]. Natural maize inbred lines exhibit variations in the Na^+^ content of their aboveground parts and in their tolerance to saline and alkaline conditions (NaHCO_3_). Specifically, a genome-wide association study revealed that the *ZmNSA1* gene influenced alterations in the Na^+^ content of the aboveground parts of plants under NaHCO_3_ treatment. Concurrently, NMT was employed to monitor changes in Na^+^ and H^+^ transmembrane transport, further elucidating the role of the *ZmNSA1* gene in plant saline-alkali tolerance [52]. The plasma membrane (PM) H^+^-ATPase (AHA) provides energy for critical physiological and biochemical processes in plant cells and plays a crucial role in plant growth and development. Research suggests that calcineurin B-like protein 10 (CBL10) may participate in the regulation of AHA. The NMT results reveal that the proton influx rate in *cbl10* mutants was significantly lower than that in WT *Arabidopsis* plants when exposed to high external pH stress, indicating that CBL10 may act as an interconnected regulator coordinating plant responses to saline and alkaline stresses [53]. Similarly, the calcium-binding protein TaCCD1 in wheat is crucial for regulating the PM H^+^-ATPase-mediated alkaline stress response. The NMT results show that *CCD1-OE* lines exhibited a significantly higher H^+^ efflux rate compared to WT, while *CCD1-RNAi* lines displayed a markedly lower rate, indicating that TaCCD1 enhances proton extrusion to mediate alkaline stress adaptation [54].

Early steps in the endoplasmic reticulum (ER) lumen and cis-Golgi involve the trimming of N-glycans by Class I α-mannosidases (MNSs), which play critical roles in root growth and stress responses. Under normal pH 6.0 conditions, the *mns1 mns2 mns3* *Arabidopsis* mutant exhibited proton influx in the elongation zone of root tips, whereas alkaline pH 8.2 induced a switch to proton efflux, indicating that alkaline treatment triggers a reversal in the proton flux direction from influx to efflux. In this study, NMT revealed that alkaline stress promotes H^+^ efflux in mutant roots, providing indirect evidence that N-glycosylation influences IAA synthesis and transport [55]. Rapeseed (*Brassica napus* L.), a widely cultivated oilseed crop, is susceptible to ion toxicity under saline-alkali conditions. NMT analysis revealed that foliar application of 0.5% CaCl_2_ solution under NaHCO_3_ stress significantly alleviated membrane damage caused by ion imbalance, suppressed Na^+^ influx and K^+^/Ca^2+^ efflux in mesophyll cells, and maintained K^+^/Na^+^ homeostasis across tissues, thereby promoting seedling growth and development [56]. Along with NMT, elucidating the mechanism of pH homeostasis regulation in plants under alkaline stress will contribute to addressing the critical issues of soil alkalization and high-pH-induced nutrient deficiency in agricultural production and ecosystems (Table 1).

### 3.3. Water Stress

Water stress is detrimental to plant growth, and the investigation into enhancing plant adaptability to water scarcity and drought stress has garnered significant attention in recent years [12,99,100]. Maintaining root growth during the adaptation process to drought-affected soil is crucial for plants [101,102]. NMT and other methods have confirmed that, under moderate water stress, plants require auxin to stimulate proton secretion at the root tip, thereby regulating the abscisic acid-mediated adaptive processes of plants in response to drought stress [57]. Furthermore, the absorption and transport of K^+^, H^+^, and Ca^2+^ play a pivotal role in the drought tolerance of crops adapted to arid agricultural environments. MIFE results reveal significant differences between long-term K^+^, H^+^, and Ca^2+^ fluxes under drought conditions and those under short-term PEG-induced drought stress. The substantial K^+^ efflux, apoplastic pH alkalinization (H^+^ influx), and early Ca^2+^ efflux response in the mesophyll may serve as chemical signals and critical indicators of drought stress intensity in soybean plants [58]. By employing NMT, the flow rates of K^+^, H^+^, and Ca^2+^ were measured within a 24 h period across various barley lines, including XZ5, a silent line, and an overexpression line. These measurements revealed the significant roles of two key K^+^ transporters, HvAKT2 and HvHAK1, in the drought tolerance of barley [59]. Drought is one of the primary limiting factors leading to reduced yield and quality in tea production. NMT and plant physiological experimental results reveal that higher ROS scavenging capacity and PM H^+^-ATPase activity are the main reasons for the superior mesophyll K^+^ retention capacity of drought-tolerant tea cultivars compared to drought-sensitive cultivars under drought stress [60].

Whole-genome duplication (or polyploidization) events may enhance plant adaptability to harsh environments. Using NMT to measure K^+^ transport rates in the guard cells of transgenic and wild-type cotton under long-term water stress revealed that GhCIPK6D1 weakens drought resistance by positively regulating K^+^ uptake, whereas GhCIPK6D3 enhances drought resistance by promoting K^+^ efflux. This reveals the functional differentiation mechanism of these genes in regulating stomatal movement [61]. Moreover, soil waterlogging creates a hypoxic environment in the root zone, severely affecting plant growth and productivity. Using MIFE, researchers investigated the K^+^ transport rate in wheat roots under hypoxic stress. The results reveal that hypoxia-sensing in tolerant cultivars triggers early accumulation of reactive oxygen species (ROS), which subsequently activates K^+^ efflux channels. This leads to decreased cytosolic K^+^ levels, ultimately inducing caspase-like protease-mediated programmed cell death (PCD). Conversely, elevated K^+^ levels inhibit caspase-like protease activity, thereby enhancing plant hypoxia tolerance. This study provides novel insights for plant breeders to improve crop waterlogging resistance by modulating K^+^ homeostasis mechanisms [62].

Auxin biosynthesis and transport are implicated in drought stress response [31]. NMT enables effective real-time monitoring of IAA transport in plant surface tissues while allowing simultaneous observation with existing live imaging techniques [103]. NMT was used with the newly developed IAA sensor to detect real-time auxin transport within rice roots under drought stress. The results reveal that the interaction between RoLe1 and OsAGAP disrupts OsAGAP function, altering polar auxin transport in the root system. This leads to reduced IAA influx into root tip cells, thereby modulating root system development and enhancing drought resistance in rice [12]. In *Poncirus trifoliata*, phenotypic analysis and NMT demonstrated that seedlings inoculated with the arbuscular mycorrhizal fungus (*Funneliformis mosseae*) exhibited significantly higher root hair density, length, diameter, and root IAA levels while displaying lower total root IAA efflux. These adaptations collectively enhance the drought resistance of host plants [63].

N^6^-methyladenosine (m^6^A) is the most prevalent internal modification in mRNAs, and drought response is highly regulated at the genomic, transcriptional, and post-transcriptional levels. In cotton, Virus-Induced Gene Silencing (VIGS) of Ca^2+^-related genes, namely *GhECA1* and *GhCNGC4*, reduced the drought resistance of cotton, which was accompanied with a decrease in the enrichment of m^6^A on the silenced genes, leading to alterations in the Ca^2+^ content [64]. Hydrogen sulfide (H_2_S) is a novel gas signal molecule that can enhance plant drought resistance by inducing stomatal closure while simultaneously improving photosynthetic efficiency. Under drought stress, the fluxes of Cl^−^, K^+^, and H^+^ in the guard cells of Chinese cabbage were detected using NMT. The results show that H_2_S signaling induced the transmembrane efflux of Cl^−^ and H^+^ while inhibiting K^+^ influx, indicating that Cl^−^ channels serve as the primary responders for H_2_S-mediated stomatal movement regulation [65]. This research underscores the critical roles of m^6^A modification and gas signal molecules such as H_2_S in plant drought response and provides insights into potential drought-tolerant strategies for crop improvement. Integrated with NMT, elucidating the physiological dynamics and molecular regulatory networks underlying plant water stress responses will contribute to addressing the increasing normalization of agricultural drought under global climate change (Table 1).

### 3.4. Low- and High-Temperature Stress

Low temperature constitutes a pivotal environmental constraint that hinders plant growth and agricultural productivity [66,102]. Rice, being a low-temperature-sensitive crop, is restricted to specific climatic zones for cultivation. Research has demonstrated the significant role of calmodulin B-like interacting protein kinases (CIPKs) under low-temperature stress conditions. Specifically, a point mutation in *OsCIPK7* in rice leads to a conformational alteration in the activation loop of its kinase domain, subsequently augmenting protein kinase activity and enhancing plant cold tolerance. Furthermore, NMT has elucidated the correlation between rice cold tolerance phenotypes and alterations in Ca^2+^ flux rates, indicating that enhanced cold tolerance in rice is associated with an augmented Ca^2+^ influx capacity [67]. Moreover, human selection for japonica rice varieties has facilitated their adaptation to low-temperature environments. The molecular basis of this adaptability is intimately linked to *COLD1*, a quantitative trait locus (QTL) associated with cold tolerance in japonica rice. The overexpression of *COLD1^jap^* markedly improves plant cold tolerance, whereas rice lines with *COLD1^jap^* deletions or downregulation exhibit increased sensitivity to low temperatures. NMT analysis of Ca^2+^ flux rates in rice roots under cold stress further validates that COLD1 interacts with G protein α, activates Ca^2+^ channels in response to low temperatures, accelerates G protein GTPase activity, and thereby enhances cold tolerance in japonica rice [8].

Additionally, OsCNGC9, a cyclic nucleotide-gated channel, augments cold tolerance in rice by modulating cold-induced Ca^2+^ influx and the subsequent elevation of cytosolic calcium levels, as detected using NMT [68]. Analogously, in cucumber, CsGPA1 interacts with CsCOR413PM2, a plasma membrane-localized protein. The inhibition of either CsGPA1 or CsCOR413PM2 results in decreased Ca^2+^ influx under low-temperature conditions, further suppressing the expression of *CsICE* and *CsCBF*. These findings provide a foundation for future investigations into the mechanism underlying cold tolerance mediated by the Gα subunit in cucumber [69]. In the Columbia ecotype of *Arabidopsis thaliana*, regardless of whether the plants are grown at 15 °C or 25 °C, the roots produce cells at the same rate and maintain consistent growth zone lengths. An analysis of whole-root oxygen consumption rates revealed that the meristematic zone exhibits steady-state Q_10_ values ranging between 0.7 and 2.1, whereas the elongation zone demonstrates higher Q_10_ values of 2.6 to 3.3, indicating that the metabolic cost of rapid cell elongation significantly exceeds that of cell division [70].

Heat stress constitutes a significant environmental challenge impacting crop growth and productivity [71,104]. The *HTS1* gene encodes a β-ketoacyl carrier protein reductase (KAR) localized in the thylakoid membrane, participating in de novo fatty acid biosynthesis. In comparison with WT plants, *hts1* mutants exhibit elevated heat-induced accumulation of H_2_O_2_, increased Ca^2+^ influx in mesophyll cells, and exacerbated membrane and chloroplast damage. This underscores the pivotal role of *HTS1* in maintaining membrane stability, chloroplast integrity, and stress signaling, which are fundamental for heat tolerance in rice [72]. Lettuce thrives in cooler environments, and high temperatures adversely affect its yield and quality. According to NMT results, exogenous spermidine augments the concentrations of Ca^2+^, K^+^, Fe^3+^, Mn^2+^, Zn^2+^, and NO_3_^−^ in lettuce leaves. It also facilitates K^+^ efflux, enhances Ca^2+^ influx, and diminishes the relative stomatal aperture under high-temperature stress. This suggests that exogenous spermidine mitigates lettuce damage induced by high-temperature stress by modulating the ion content and altering stomatal morphology [73].

The phenomenon of global warming poses a threat to crop production. A natural quantitative trait locus, designated as TT2 (THERMOTOLERANCE 2), encodes a Gγ subunit that confers heat tolerance to rice during both vegetative and reproductive growth stages without compromising yield. The disruption of TT2 leads to a heat-triggered reduction in Ca^2+^, which weakens the interaction between the transcription factor SCT1 (Sensing Ca^2+^ Transcription Factor 1) and calmodulin. This, in turn, influences heat tolerance and variations in the waxy content in rice [74]. Furthermore, long non-coding RNAs (lncRNAs) play a role in plant stress responses. Among the heat-responsive lncRNAs in *Populus simonii*, TCONS_00202587 binds to upstream sequences via its secondary structure, interfering with target gene transcription. Additionally, using NMT, TCONS_00260893 enhances Ca^2+^ influx following heat treatment by disrupting specific variants or isoforms of the target gene. These observations indicate that lncRNAs can regulate their target genes by functioning as potential RNA scaffolds or through RNA interference pathways [75]. Integrated with NMT, elucidating the physiological regulatory networks and molecular signaling pathways of extreme temperature stress responses in plants will contribute to addressing agricultural challenges posed by the increasing frequency of extreme high/low temperatures under global climate change (Table 1).

### 3.5. Nutrition Stress

Nitrogen (N) and phosphorus (P) constitute indispensable macronutrients for plant growth and development. In seedlings of the dwarfing rootstock “M9-T337”, N or P deficiency, when compared to conditions of adequate nutrient supply, suppressed aboveground growth. Additionally, such deficiencies augmented the partitioning of total N and P contents towards the root system [76]. Consequently, there was an increase in the total number of root tips, root length, root volume, and root surface area, accompanied by an elevation in the root-to-shoot ratio. Furthermore, both phosphorus deficiency and nitrogen deficiency hindered the influx of NO_3_^−^ into the root system, with H^+^ pumping playing a pivotal role in the plant’s response to these deficiencies [76]. In wheat, NMT results reveal that low N stress promotes lateral root development and nitrogen assimilation by regulating plant hormone signaling (such as increasing IAA), enhancing H^+^-ATPase activity and H^+^ efflux to expand N uptake capacity. This adaptation elevates the activity of key enzymes (nitrate reductase, glutamine synthetase, and glutamate synthase), stimulates protein synthesis, and drives root growth under N-deficient conditions [77]. Modern semi-dwarf rice varieties, which are a hallmark of the “Green Revolution”, necessitate considerable quantities of N fertilizers to attain high yields. The interaction between strigolactones and gibberellins is advantageous in modulating the adaptation of rice root metabolism and development under low-N conditions, thereby ensuring the efficient absorption and translocation of available nitrogen. This synergistic effect facilitates the formulation of strategies aimed at enhancing nitrogen use efficiency in high-yielding crops [78].

Understanding the physiological processes underlying N assimilation sheds light on how boreal coniferous ecosystems develop adaptation strategies to their environmental conditions. The study revealed that white spruce roots exhibited the highest N uptake and proton efflux near the root tip, with both fluxes decreasing gradually at greater distances from the tip under treatment with 50 μM of N. In contrast, exposure to 1500 μM of N triggered significant ammonium (NH_4_^+^) efflux in certain root segments, highlighting differential physiological responses to varying nitrogen availability [79]. Moreover, ectomycorrhizal (EM) roots of lodgepole pine exhibited net NH_4_^+^ uptake, while nonmycorrhizal roots showed NH_4_^+^ efflux, with EM-associated seedlings displaying a higher N content in the roots and shoots, particularly when colonized by *Laccaria bicolor*. The study revealed EM fungi’s role in reducing futile NH_4_^+^ cycling and demonstrated lodgepole pine’s preference for NH_4_^+^ over NO_3_^−^, with NH_4_^+^ uptake rates increasing under NH_4_^+^-starved conditions [80].

Iron (Fe) deficiency exerts a profound impact on the growth, development, fruit productivity, and overall quality of apples. In response to Fe deficiency stress, apple roots exhibit an adaptive mechanism by augmenting the secretion of H^+^, ultimately leading to soil acidification. The phosphorylation process, facilitated by the MAP kinase MxMPK6-2, exerts both direct and indirect regulatory effects on the activity of the plasma membrane H^+^-ATPase MxHA2. This regulation occurs at both the post-translational and transcriptional levels, thereby synergistically intensifying H^+^ secretion and enhancing root acidification in apple rootstocks subjected to Fe deficiency stress [29]. Additionally, under Fe deficiency conditions in the apple rootstock *Malus xiaojinensis*, the induced kinase MxMPK4-1 demonstrates a synergistic interaction with the IQ motif-containing protein 3 (MxIQM3). This interaction forms a functional complex that actively modulates the activity of the plasma membrane H^+^-ATPase during the Fe deficiency response [81]. Aquaporins are indispensable transmembrane proteins responsible for the transport of water and several neutral solutes. In cassava, the targeted knockdown of the aquaporin gene *MePIP2;7* results in magnesium (Mg) deficiency in basal mature leaves, manifested by yellowing and necrotic spots at the leaf edges, accompanied by excessive starch accumulation. Protein interaction studies have elucidated that *MePIP2;7* is implicated in Mg^2+^ absorption and transport through its interaction with the low-affinity Mg^2+^ transporter MeMGT9 [105]. Along with NMT, deciphering the physiological and molecular response mechanisms to plant nutrient stress provides solutions for soil nutrient imbalance or deficiency in agroecosystems (Table 1).

### 3.6. Ammonium Toxicity and Acid Stress

N is an essential macronutrient for plant growth, with NH_4_^+^ and NO_3_^−^ serving as the primary inorganic N sources utilized by plants. However, when NH_4_^+^ becomes the dominant nitrogen source, plants exhibit severe toxicity symptoms. For instance, the inhibition of root growth is one of the hallmark manifestations of NH_4_^+^ toxicity in plants [106,107]. High NH_4_^+^ levels impair wheat culm strength, vascular bundle integrity, nitrogen remobilization, and grain filling by competitively inhibiting K^+^ uptake, but supplemental K^+^ alleviates these effects by restoring transmembrane K^+^ influx and tissue K^+^ contents. This study links NH_4_^+^ toxicity in wheat to disrupted cation balance, demonstrating K^+^ supplementation as a mitigation strategy for maintaining structural and physiological functions under excessive NH_4_^+^ [82].

The nitrate transporter NRT1.1 is involved in mediating the effects of NH_4_^+^ toxicity. In *Arabidopsis*, nitrate transporter NRT1.1 exacerbates NH_4_^+^ toxicity by enhancing NH_4_^+^ uptake (potentially measurable via NMT) and disrupting assimilation, leading to ethylene-driven senescence, while *NRT1.1* mutants mitigate toxicity through improved NH_4_^+^ metabolism and reduced accumulation [83]. Moreover, the nitrate transporter NRT1.1 interacts with the nitrate efflux channel SLAH3 to form a functional unit, which alleviates NH_4_^+^ toxicity by coordinating NO_3_^−^ transport and balancing rhizosphere pH via H^+^ uptake regulation, as demonstrated using NMT to monitor H^+^ flux under high-NH_4_^+^/low-NO_3_^−^ conditions [84]. Along with NMT, clarifying the physiological and molecular mechanisms of NH_4_^+^ toxicity in plants can help address the excessive accumulation of NH_4_^+^ in agricultural soils (Table 1).

H^+^ in acidic soil can hinder plant growth [108]. However, the mechanism by which plants optimize their biological processes to reduce the adverse effects of H^+^ stress still needs further exploration. Tea (*Camellia sinensis*) plants grow in acidic soil, and NMT revealed that Fe-sufficient conditions enhanced acidic stress tolerance in tea plants by promoting Fe plaque formation on the roots, which increased plasma membrane H^+^-ATPase activity and H^+^ efflux at pH 4.0–5.0, ultimately improving N accumulation compared to pH 6.0 conditions. The findings demonstrate that Fe plaque-mediated H^+^-ATPase activation, quantified through NMT measurements of proton flux, is a key mechanism underlying tea plant adaptation to acidic environments [85]. In *Arabidopsis thaliana*, the transcription factor STOP1 enhances low pH tolerance by directly activating *NRT1.1* expression, which increases nitrate uptake to improve N use efficiency and reduces rhizospheric H^+^ levels, thereby promoting root growth in acidic soils. The STOP1-NRT1.1 module optimizes plant adaptation to acidic stress by coordinating nitrate transport and H^+^ homeostasis [86].

Climate change brings alternating patterns of severe drought and intense flooding events. These waterlogged conditions induce cytoplasmic acidification through oxygen deprivation in plant cells, ultimately inhibiting vital biological processes in plants. NMT and molecular biology experimental results demonstrate that *Arabidopsis* S-type anion channel AtSLAH3 directly senses cytosolic acidosis via the protonation of histidine residues, triggering structural activation and anion efflux to mediate flood stress tolerance, with wild-type plants outperforming *slah3* mutants under flooding conditions [87]. Aluminum (Al), a common element in the Earth’s crust, negatively impacts vegetation in acidic soils by impairing root system expansion and hindering normal plant growth processes [109]. Soil acidification in apple orchards leads to the release of root-toxic Al^3+^ into the soil. Melatonin can alleviate Al toxicity in apple roots by activating the MdSTOP1-MdNAC2 transcriptional complex, which upregulates *MdALS3* and *MdNHX2* to enhance vacuolar H^+^/Al^3+^ exchange and H^+^ homeostasis, with NMT confirming the ion flux dynamics critical for Al stress mitigation [88]. Interestingly, an appropriate Mg supply can alleviate the toxic effects of high-concentration Al on poplar root growth. Al toxicity inhibits polar auxin transport and distribution in the root transition zone, but Mg supplementation partially mitigates this effect. Further analysis using NMT on the auxin transporter mutant *pin-formed2* (*pin2*) revealed that Mg alleviates Al toxicity by regulating root surface alkalinization in the transition zone through PIN2-mediated polar auxin transport [89]. Along with NMT, resolving the physiological dynamics and molecular regulatory networks of plant acid stress responses provides theoretical foundations for addressing crop growth inhibition, root developmental disorders, and nutrient imbalance caused by H^+^ toxicity in acidic soils (Table 1).

### 3.7. Heavy Metal Toxicity

Heavy metals constitute a significant category of environmental contaminants [90]. Upon exposure to heavy metals, plants undergo alterations in morphogenesis, cell membrane permeability, photosynthesis, respiratory metabolism, enzymatic processes, and genetic impacts. When the concentration of heavy metals surpasses the threshold tolerance level of plants, it induces toxicity, disrupts metabolic processes, and inhibits plant growth [110]. Cadmium (Cd) is a particularly prominent pollutant in agricultural land, which not only severely restricts crop production but also poses a grave risk to human health through bioaccumulation in the food chain [2]. The investigation revealed that female *Populus cathayensis* exhibited heightened Cd absorption and translocation from roots to aboveground parts, whereas male *Populus cathayensis* demonstrated substantial Cd accumulation in roots, enhanced antioxidant capacity, and the effective sequestration of Cd within cells and bark. Furthermore, NMT was employed to monitor the Cd absorption rate in *Populus cathayensis* roots. The results indicate that the net Cd^2+^ influx in female *Populus cathayensis* was greater than that in male individuals, suggesting that male *Populus cathayensis* possesses higher Cd tolerance, thereby offering novel insights into the potential mechanisms underlying gender-specific responses to Cd stress [91]. NMT and biochemical experimental results confirm that in *Populus euphratica*, calcium-dependent protein kinase 21 (PeCPK21) interacts with the *Arabidopsis* nuclear transcription factor YC3 (AtNF-YC3) to reduce Cd accumulation and enhance the ROS scavenging system, thereby positively regulating the plant’s adaptive capacity to Cd-contaminated environments [92].

*Eichhornia crassipes* is an effective ecological remediation plant that can alleviate Cd stress. Under prolonged Cd stress treatment, NMT was employed to measure the transport rates of O_2_ and H_2_O_2_ in the roots and leaves of *Eichhornia crassipes* under stress induced by treatment with 4 mg L^−1^ CdCl_2_. The results show that, compared to the control, the Cd treatment significantly inhibited the O_2_ uptake rates in both the roots and leaves, markedly enhanced H_2_O_2_ efflux in the roots, and suppressed H_2_O_2_ efflux in the leaves. This indicates that Cd stress suppresses cellular respiration in the roots and leaves and disrupts ROS homeostasis in *Eichhornia crassipes* [93]. Additionally, NMT was utilized to measure the Cd^2+^ flux rate on the root surface of Chinese cabbage, revealing that hemin treatment reduced Cd accumulation in Chinese cabbage seedlings by decreasing plant Cd absorption rather than by influencing Cd translocation within the plant [94]. Analogously, NMT provided direct evidence that silicon (Si) treatment can enhance Cd tolerance in marine diatoms and maintain their metal homeostasis [95]. In barley, chloride (Cl^−^) enhances Cd mobility and phytotoxicity by increasing Cd^2+^ uptake and disrupting ion homeostasis in roots, with Cd-sensitive genotypes exhibiting higher Cl^−^-mediated Cd accumulation and photosynthetic impairment. The findings underscore the critical role of soil Cl^−^ in Cd toxicity and propose breeding low-Cl^−^-uptake barley varieties to mitigate Cd contamination risks, supporting safer agricultural production for global food and beverage industries [96]. Regarding stress mitigation analysis, NMT revealed that overexpression of *PeANN1* (an annexin encoding gene facilitating Cd enrichment) enhances Cd^2+^ accumulation in transgenic *Arabidopsis*, and it can serve as a candidate gene for phytoremediation to alleviate cadmium stress [97]. In apple rootstocks, the NMT and plant physiology experiment results showed that exogenous melatonin reduces Cd accumulation in the aboveground parts of apple plants and alleviates Cd toxicity. This effect is likely attributed to melatonin-mediated compartmentalization of Cd within tissues, as well as its induction of the antioxidant defense system and upregulation of key genes involved in detoxification-related transcriptional regulation [98]. When integrated with NMT, analyzing ion transport dynamics and the detoxification of regulatory pathways under heavy metal stress addresses escalating ecological risks from soil contamination in mining/agricultural areas (Table 1).

## 4. Conclusions and Prospects

**Figure 2:**
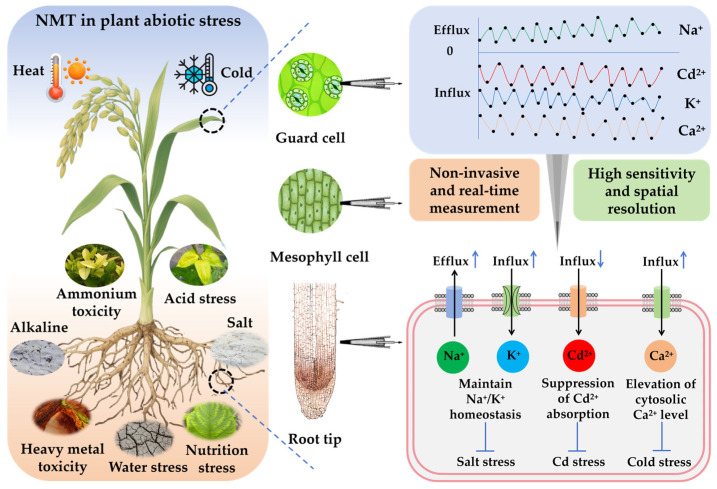
A comprehensive model of NMT application in elucidating plant responses to abiotic stress conditions. This schematic illustrates the integration of NMT with various major abiotic stress types, including but not limited to water stress, salt stress, alkali stress, extreme temperature stress, ammonium toxicity, acid stress, heavy metal toxicity, and nutrient imbalance. The model demonstrates how NMT can be used to monitor real-time dynamic fluxes (influx and efflux) of different ions (e.g., Na^+^, K^+^, Cd^2+^, and Ca^2+^) within different plant cells and tissues (e.g., guard cell, mesophyll cell, and root tip), thereby revealing the stress defense mechanisms of plants. By non-invasive, real-time, high sensitivity and spatial resolution measuring ion transport rate, this model provides a deeper mechanistic understanding of plant adaptation strategies and stress signaling pathways. The circles represent the measurement points for NMT. Black arrows indicate the direction of ion flux. Blue arrows represent increases or decreases in the magnitude of ion influx or efflux. Blue blunt arrowheads represent defense responses against abiotic stress.

The application of NMT in plant stress physiology has reached substantial maturity, providing a comprehensive platform for ion/molecule flux analysis that significantly advances our understanding of plant stress resistance mechanisms. NMT should be used to study major abiotic stress types (e.g., water stress, salt stress, alkali stress, extreme temperature stress, ammonium toxicity, acid stress, heavy metal toxicity, and nutrient imbalance) to monitor real-time dynamic fluxes (influx and efflux) of different ions (e.g., Na^+^, K^+^, Cd^2+^, and Ca^2+^) within specific plant cells and tissues (e.g., guard cell, mesophyll cell, and root tip). This non-invasive approach with high sensitivity and spatial resolution enables the elucidation of plant stress defense mechanisms and provides deeper insights into plant adaptation strategies and stress signaling pathways (Figure 2).

Abiotic stress effects are dynamic and influenced by factors such as plant age, genotype, stress duration, and application methods. NMT addresses this challenge through several technical advantages. Its non-invasive nature allows for repeated measurements on the same sample over extended periods without causing tissue damage. The system incorporates environmental control chambers that maintain stable measurement conditions (humidity, temperature, and CO_2_) during prolonged experiments. For genotype/age comparisons, we recommend using standardized protocols including synchronized plant growth stages, controlled stress application gradients, and multiple biological replicates. Furthermore, regarding stress mitigation analysis, NMT can quantitatively evaluate the efficacy of mitigation strategies (e.g., osmoprotectants) by comparing pre-/post-treatment ion flux patterns. NMT detects early recovery signatures (e.g., K^+^ flux restoration in roots post-drought) before visible phenotypic recovery occurs. And spatial resolution enables a localized analysis of mitigation effects, such as differentiated responses in root apical and mature zones.

However, some critical challenges persist in optimizing this technology, such as hypersaline soils (inducing signal noise from competing ions like Na^+^/Cl^−^), severe heavy metal contamination (e.g., Cd^2+^ saturation overwhelming sensor resolution), and temperature extremes (altering membrane integrity and flux kinetics). For instance, in acidic soils, H^+^ efflux measurements may conflate abiotic proton gradients with biologically regulated fluxes, while freezing conditions disrupt microelectrode stability. These limitations risk overlooking transient but biologically significant signaling events during stress adaptation. Moreover, the reliability of NMT data hinges on rigorous sample preparation and environmental control, as subtle discrepancies in these steps can profoundly affect reproducibility. For example, sample preparation variability, including root hair density differences and microelectrode positioning accuracy, could induce flux measurement deviations. The inconsistent growth medium composition (e.g., Ca^2+^ or chelator concentrations) could unpredictably modify ion availability and membrane potential dynamics, complicating data analysis. Environmental control parameters, such as temperature fluctuations and solution convection artifacts, may cause error interference in the measurement and analysis of NMT data.

Looking forward, some strategic developments are promising in transforming NMT applications. Its integration with multi-omics platforms could enable unprecedented mechanistic insights, for instance, correlating Ca^2+^ flux dynamics with stress-responsive gene clusters (e.g., SOS pathway genes, auxin transport, and response factors) identified through single-cell RNA sequencing or validating ABA-mediated stomatal regulation through parallel proteomic profiling of guard cell H^+^-ATPases. Artificial intelligence implementation may show promise in two operational domains: machine vision algorithms could automate microelectrode navigation with submicron precision, and deep learning models trained on historical flux datasets may predict stress response thresholds, effectively reducing human interpretation bias.

In conclusion, NMT possesses considerable potential to occupy a central position in agricultural breeding endeavors and ecological environment preservation. It can provide scientific underpinnings for the cultivation of stress-tolerant crops, the enhancement of ecological conditions, and the attainment of sustainable development objectives.

## Figures and Tables

**Table 1:** NMT application in plant abiotic stress.

Stress	Samples	Detection Site	Ions/Molecules	References
	Rice	Seed embryo	K^+^, Na^+^	[36]
	Quinoa	Root elongation and mature zone	K^+^	[37]
	Quinoa	Leaf mesophyll cells	Ca^2+^, K^+^, H^+^	[38]
	Barley and triticale	Root elongation and mature zone	Ca^2+^, K^+^, H^+^	[39]
	Rice	Root mature zone	K^+^, Ca^2+^	[40]
	*Arabidopsis*	Root meristematic zone	Na^+^	[41]
	*Arabidopsis*	Roots	Na^+^, H^+^	[42]
	*Populus euphratica*	Roots	Na^+^	[43]
	*Populus euphratica*	Root meristematic zone	Na^+^	[44]
	*Limonium bicolor*	Salt glands	Na^+^	[45]
	*Nitraria tangutorum*	Root tip	Na^+^, K^+^	[46]
	Sugar beet	Roots	Cl^-^	[47]
	Barley	Roots	K^+^, H^+^	[48]
	*Kandelia obovata*	Leaves	Na^+^	[49]
	*Kandelia obovata*	Roots	Na^+^, K^+^, H^+^, Ca^2+^	[50]
	*Kandelia obovata*	Roots	Na^+^, K^+^	[51]
Alkali stress	Maize	Root meristematic zone	Na^+^, H^+^	[52]
	*Arabidopsis*	Leaf mesophyll cells	H^+^	[53]
	Wheat	Roots	H^+^	[54]
	*Arabidopsis*	Root elongation zone	H^+^	[55]
	Rapeseed	Leaf mesophyll cells	Na^+^, K^+^, Ca^2+^	[56]
Water stress	Upland rice	Root tip	IAA	[12]
	Rice, *Arabidopsis*	Root tip	H^+^	[57]
	Soybean	Leaf mesophyll cells	K^+^, H^+^, Ca^2+^	[58]
	Barley	Leaf mesophyll cells, roots	K^+^, H^+^, Ca^2+^	[59]
	Tea	Roots	K^+^	[60]
	Cotton	Guard cell	K^+^	[61]
	Barley	Roots	K^+^	[62]
	Trifoliate orange	Root hair zone	IAA	[63]
	Cotton	Leaf mesophyll cells	Ca^2+^	[64]
	Chinese cabbage	Guard cell	Cl^−^, K^+^, H^+^	[65]
Low-temperature stress	Rice	Root meristematic zone	Ca^2+^	[8]
	Watermelon	Intracellular	Ca^2+^	[66]
	Rice	Roots	Ca^2+^	[67]
	Rice	Roots	Ca^2+^	[68]
	Cucumber	Roots	Ca^2+^	[69]
	*Arabidopsis*	Roots	O_2_	[70]
High-temperature stress	*Arabidopsis*	Leaf mesophyll cells	H^+^, K^+^, Ca^2+^	[71]
	Rice	Leaf mesophyll cells	Ca^2+^	[72]
	Lettuce	Guard cell	K^+^, Ca^2+^	[73]
	Rice	Root and aboveground parts	Ca^2+^	[74]
	Poplar	Roots	Ca^2+^	[75]
Nutrition stress	Apple	Stock root elongation zone	H^+^	[29]
	Apple	Stock roots	H^+^, NO_3_^−^	[76]
	Wheat	Roots	IAA, H^+^	[77]
	Rice	Root meristem zone	NH_4_^+^	[78]
	White spruce	Roots	H^+^, NH4^+^, NO_3_^−^	[79]
	Lodgepole pine	Root and aboveground parts	H^+^, NH4^+^, NO_3_^−^	[80]
	Apple rootstock	Root mature zone	H^+^	[81]
Ammonium toxicity	Wheat	Roots	K^+^	[82]
	*Arabidopsis*	Roots	NH4^+^, NO_3_^−^	[83]
	*Arabidopsis*	Roots	NO_3_^−^	[84]
Acid stress	Tea	Root mature area	H^+^	[85]
	*Arabidopsis*	Root meristem zone, elongation zone and mature zone	H^+^	[86]
	*Arabidopsis*	Roots	Cl^−^, NO_3_^−^	[87]
	*Malus hupehensis*	Roots	H^+^	[88]
	Populus	Roots	Mg^2+^, IAA	[89]
Heavy metal toxicity	*Sedum plumbizincicola*	Roots	Cd^2+^	[90]
	Cathay poplar	Roots	Cd^2+^	[91]
	*Populus euphratica*	Root tip	Cd^2+^	[92]
	*Eichhornia crassipes*	Roots, stem, leaves	H_2_O_2_, O_2_	[93]
	Pak choi	Roots	Cd^2+^	[94]
	Diatom	Frustule	Cd^2+^	[95]
	Barley	Roots	Cd^2+^, K^+^, H^+^, Cl^−^, Ca^2+^	[96]
	*Populus euphratica*	Roots	Cd^2+^	[97]
	Apple rootstocks	Roots	Cd^2+^	[98]

This table summarizes stress types, plant samples, detection sites, target ions/molecules, and corresponding references, highlighting NMT’s utility in monitoring ion/molecule dynamics under abiotic stress conditions. Stress types: categories of abiotic stress (e.g., salt stress, alkali stress, water stress, extreme temperature stress, nutrition stress, ammonium toxicity, acid stress, heavy metal stress). Samples: plant species or tissues analyzed (e.g., *Arabidopsis*, rice, apple). Detection site: specific plant organs or subcellular regions measured (e.g., root tips, leaf mesophyll cells). Ions/molecules: ions or signaling molecules (e.g., K^+^, Na^+^, Ca^2+^, NH_4_^+^, H^+^, Cd^2+^, NO_3_^−^, IAA) tracked using NMT. References: key studies demonstrating NMT’s application in abiotic stress physiology.
